# 2019 List of Reviewers

**DOI:** 10.1117/1.NPh.7.1.010101

**Published:** 2020-01-17

*Neurophotonics* would like to sincerely thank the following individuals who served as reviewers in 2019. The success of our publication hinges on the voluntary contributions of time and energy put forth by these professionals.

Yama Akbari
Anna Letizia Allegra Mascaro
Maria Arredondo
David Atchison
Jaideep Bains
Wesley Baker
Matilde Balbi
Gemma Bale
Adam Bauer
Bernhard Baumann
Lisa Beckman
Lisa Beckmann
Ken Berglund
Jason Berwick
Irving Bigio
Pablo Blinder
David Boas
Heather Bortfeld
Sabrina Brigadoi
Ralf Brinkmann
Erin Buckley
Stefan Carp
Paolo Cassano
Siyu Chen
Xiaojun Cheng
Mykyta Chernov
Carol Y. Cheung
Antonio Chiarelli
Adam Cohen
Emma Condy
Irene Costantini
Verginia Cuzon Carlson
Jerry Dadap
Irene de la Cruz Pavia
Hamid Dehghani
Anna Devor
Ilka Diester
Patrick Drew
Adam Eggebrecht
Christian Eggeling
Rinat Esenaliev
Zoltan Fekete
Rodrigo Forti
Dan Fu
Tsukasa Funane
Tamas Fuzesi
Jessica Gemignani
Michael Giacomelli
Greg Giannone
Ariel Gilad
Yukiori Goto
Michael Hamblin
Danielle Harper
Joy Hirsch
Fumitaka Homae
Keum-Shik Hong
Jue Hou
Zhihong Hu
Xiangrun Huang
Daniel Huber
Jennifer Hunter
E. Duco Jansen
Huabei Jiang
Kivilcim Kilic
Beop-Min Kim
Chulhong Kim
Joseph La Manna
Hsinyi Lai
Frederic Lange
Kirill Larin
Francesco LaRocca
Frederic Lesage
Lei Li
Peng Li
Adam Liebert
David Liebeskind
Chao Liu
Hanli Liu
Ting Luo
Hongtao Ma
Nicholas MacKinnon
Ryan McNabb
Yukifumi Monden
Christopher Moore
Yusuke Moriguchi
Timothy Murphy
Noman Naseer
Kamran Nasiriavanaki
Thien Nguyen
Ruiqing Ni
Konstantin Nikolic
Haijing Niu
Sergio Novi Junior
Jonathan Nylk
Rodney O’Connor
Marco Pagliazzi
B. Hyle Park
Ashwin Parthasarathy
Stephane Perrey
Shaohua Pi
Daqing Piao
Paola Pinti
Laura Pirazzoli
Filippo Pisano
Philippe Pouliot
Jun Qian
Ajay Rajaram
Anna Roe
John Rogers
Caroline Runyan
Sava Sakadzic
Michelle Sander
Hendrik Santosa
Hiroki Sato
Christopher Schaffer
Felix Scholkmann
Fei Shi
Andy Shih
Pavel Shilyagin
Sotaro Shimada
Shy Shoham
Alejandra Sierra Lopez
Ludovico Silvestri
Garth Simpson
Weiye Song
John Spencer
Vivek Srinivasan
Colin Sullender
Jason Sutin
Stephanie Sutoko
Nick Swindale
Ilias Tachtsidis
Toshimitsu Takahashi
Stefan Talu
Johnny Tam
Jianbo Tang
Qinggong Tang
Martin Thunemann
Jan Tonnesen
Peter Torok
Antoine Triller
Kotaro Tsuboi
Daisuke Tsuzuki
Vassiliy Tsytsarev
Ben Urban
Matt Valley
Gregoire Vergotte
Alexander von Lühmann
Heidrun Wabnitz
Zheng Wang
Jack Waters
Timothy Weber
Wei Wei
Kit Werley
Lara Wierenga
Ian Winship
Wang Xi
Augix Xu
Guohua Xu
Ninglong Xu
Junjie Yao
Xincheng Yao
Mohammad Yaseen
Ji Yi
Zhen Yuan
Pengfei Zhang
Tingwei Zhang
Wen Zhang
Xian Zhang
Chaozhe Zhu
Bernhard Zimmermann

